# Benzoate of Ammonia in Gout

**Published:** 1847-09

**Authors:** 


*Benzoate of Ammonia in Gout.*—Dr. Seymour states that he has frequently used this medicine in cases in which the small joints were red and swollen, or where fluid was deposited in the joint of the great toe, and also in cases where the lithate of soda existed in the joints of the fingers, and that it was decidedly useful. He thinks that early depositions have been arrested, and large depositions diminished, under the use of this medicine. He regards it as a good diuretic, and as especially adapted to those cases of dropsy in which an irritable stomach renders the employment of ordinary diuretics impracticable. He has seen also the albumen in renal dropsey diminish under the use of the benzoate of ammonia.—*Ibid, from Thoughts on Several Severe Diseases, &c.*

